# Microenvironment Influence of a Novel Bioengineered Wound Product, APIS®: A Preliminary *In Vitro* Analysis of Inflammatory Marker and Growth Factor Secretion

**DOI:** 10.1155/2021/6612870

**Published:** 2021-03-20

**Authors:** Isaac Rodriguez, Tricia Conti, Nina Bionda

**Affiliations:** ^1^SweetBio, Inc., 460 S. Highland St. Floor 2, Memphis, TN 38111, USA; ^2^iFyber, LLC, 950 Danby Rd Ste 198, Ithaca, NY 14850, USA

## Abstract

**Objective:**

Preliminary biological activity assessment of a novel bioengineered wound product (*APIS®, SweetBio, Inc., Memphis, TN, USA*), a synthesis of gelatin, Manuka honey, and hydroxyapatite, with *in vitro* indications to protect, instill balance to, and progress the wound microenvironment. *Approach*. The biological activity the bioengineered wound product (BWP) elicits on human cells *in vitro* was assessed by evaluating matrix metalloproteinase- (MMP-) related proteins expressed by macrophages and secretion of growth factors in fibroblasts. Cells were cultured with no treatment, stimulated with lipopolysaccharides (LPS), or seeded directly on the BWP for 24 hours. An additional 72-hour time point for the BWP was assessed to determine if the BWP maintained its activity compared to itself at 24 hours. Cell culture supernatants were assayed to quantify secreted protein levels.

**Results:**

MMP-9 secretion from macrophages seeded on the BWP were nondetectable (*P* < 0.01), while a tissue inhibitor of MMP (TIMP-1) was detected. This decreased the overall MMP-9/TIMP-1 ratio secreted from macrophages seeded on the BWP compared to the controls. Additionally, the secretion of prohealing growth factors such as basic fibroblast growth factor (FGFb) and vascular endothelial growth factor (VEGF) was observed.

**Conclusion:**

Results from this preliminary *in vitro* evaluation suggest that the BWP has the potential to instill balance to the wound microenvironment by reducing the MMP-9/TIMP-1 ratio secretion from macrophages and progress previously stalled chronic wounds towards healing by triggering the release of growth factors from fibroblasts.

## 1. Introduction

Diabetes mellitus has become a global pandemic, with approximately 422 million people affected worldwide [1–3]. The likelihood for these patients to develop a diabetic foot ulcer (DFU) during their lifetime is up to 25% [4]. DFUs are chronic wounds that are difficult to heal because of the disruption of the normal wound healing cascade [5]. The consequences associated with nonhealing DFUs are grave. Globally, it is estimated that every 30 seconds, a leg is amputated and that 85% of these amputations are attributed to DFUs [6]. Furthermore, amputation complications are responsible for mortality rates of 13–40%, 35–65%, and 39–80% at 1, 3, and 5 years after amputation, respectively [4]. Other chronic and proinflammatory wounds, such as venous leg ulcers, pyoderma gangrenosum, and calciflaxis, also present a major biological, psychological, and financial burden on patients and the broader healthcare system [7–10]. Therefore, the need for advanced treatments to restore the normal wound healing cascade to progress chronic and proinflammatory wounds toward healing is not only vital for limb preservation but also for saving lives.

To understand how to heal chronic (and proinflammatory) wounds, it is crucial to understand the normal wound healing cascade, where it breaks down, and the criticality of the appropriate balance of inflammatory cytokines and growth factors. The normal wound healing cascade can be described as four consecutive stages: hemostasis, inflammation, proliferation, and remodeling [11]. Disruption of this cascade is linked to the development of nonhealing, chronic wounds [5]. Matrix metalloproteinases (MMPs) and tissue inhibitors of MMPs (TIMPs) are essential biomarkers for normal wound healing as they play a major role in extracellular matrix degradation [12–14]. However, elevated levels of MMPs and deflated levels of TIMPs have been correlated to a stalled or prolonged inflammatory stage, resulting in chronic wounds or wound failure [12, 14, 15]. Specifically, multiple clinical studies have analyzed fluid from chronic and acute wounds and concluded that increased MMP-9/TIMP-1 ratios are associated with poor wound healing [13, 16–19]. This proteolytic imbalance can lead to the degradation of matrix components, growth factors, and other proteins necessary for the successful completion of the healing cascade [12, 19]. Once balance to the wound microenvironment is restored, healing may progress out of the inflammatory phase to the proliferative and remodeling phases, during which growth factors play key roles in signaling cell proliferation, migration, and differentiation. These growth factors, such as basic fibroblast growth factor (bFGF), vascular endothelial growth factor (VEGF), and epidermal growth factor (EGF), are readily present in the acute wound environment but have been demonstrated to be deregulated in the chronic wound microenvironment [20, 21]. Therefore, new treatment strategies for healing chronic wounds, such as DFUs, could be directed towards instilling balance to and progressing the microenvironment by reducing concentrations of MMPs, increasing levels of TIMPs, and increasing levels of growth factors [12, 20].

**Figure 1:**
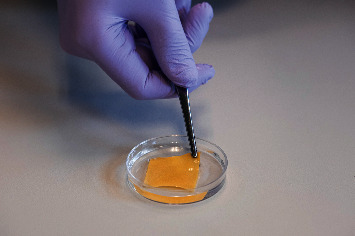
Image of the hydrated BWP.

The preliminary studies presented in this manuscript were performed to assess the *in vitro* biological activity of a novel bioengineered wound product (APIS®, SweetBio, Inc., Memphis, TN, USA). Specifically, the contribution of the bioengineered wound product (BWP) to balance and progress the chronic wound microenvironment was evaluated by analyzing the secretion of inflammatory cytokines from macrophages and growth factors from fibroblasts in an *in vitro* cell culture set up. The BWP is a solid biodegradable sheet manufactured through an advanced synthesis of gelatin (a highly purified collagen derivative), Manuka honey, and hydroxyapatite (HAp) (Figure 1). Each of these materials has well-established records of biocompatibility and biological activity with respect to wound healing. Gelatin is a natural protein matrix that is beneficial for cell adhesion, migration, and proliferation, as well as wound granulation and epithelialization [22–25]. Gelatin is also known to attract and buffer the elevated levels of MMP-2 and MMP-9 in the chronic wound microenvironment by serving as a sacrificial substrate [26]. Manuka honey is widely known for its antibacterial and anti-inflammatory properties, but it has also shown to activate growth factors, increase fibroblast activity, and suppress the expression of MMP-2 and MMP-9 [27, 28]. HAp is an inorganic mineral that can bind proteins, ions, and other molecules from the surrounding environment which can trigger the adhesion, proliferation, and differentiation of cells [29, 30]. Specifically, hydroxyapatite has been shown to promote wound healing via re-epithelialization, matrix formation, angiogenesis, and recruitment of macrophages and fibroblasts [30–34]. Considering the variety of biological activities for the three biomaterials, it was hypothesized that the BWP would elicit similar *in vitro* effects on human cells consistent with instilling balance to a stalled wound microenvironment and progressing the wound towards healing.

## 2. Materials and Methods

### 2.1. Macrophage Culturing and Seeding

Human monocytes (THP-1, ATCC TIB-202, Manassas, VA, USA) were differentiated into macrophages using an established iFyber protocol based on a procedure found in the literature [35]. Briefly, the monocytes were cultured in an RPMI medium (Sigma-Aldrich, St. Louis, MO, USA) containing 25 nM phorbol 12-myristate 13-acetate (PMA, Calbiochem, San Diego, CA, USA) for 48 hours, followed by a 24-hour rest period in the culture medium without PMA. Upon the conclusion of the rest period, the cells were prepared for seeding by washing once with sterile 1X phosphate buffered saline (PBS) and then detached from the tissue culture flask using a 0.05% trypsin/ethylenediaminetetraacetic acid solution (EDTA, Life Technologies, Carlsbad, CA, USA). PBS was comprised of 96 mM NaCl (Avantor, Radnor, PA, USA), 1.9 mM KCl (Merck KGaA, Darmstadt, Germany), 7.1 mM Na_2_HPO_4_ (Fisher Chemical, Fair Lawn, NJ, USA), and 1.2 mM KH_2_PO_4_ (MP Biomedicals, Solon, OH, USA).

Experiments were performed in a 12-well tissue culture plate (assay plate) with the following treatments assessed in triplicate (*n* = 3) at a 24-hour time point: incubation with the BWP, stimulation with lipopolysaccharides (LPS, Sigma-Aldrich, standard positive control for THP-1 cells), and no treatment (negative control). An additional 72-hour time point for the BWP was assessed to determine if the BWP maintained its activity compared to itself at 24 hours. To prepare the assay plate, wells that were to get the BWP were rinsed with 1 mL of Anti-Adherence Rinsing Solution (STEMCELL Technologies, Cambridge, MA, USA) such that cells would not inadvertently attach to the tissue culture plastic. The rinsing solution was then removed, and the wells were washed once using 1 mL sterile 1X PBS. Circular coupons of the BWP were cut aseptically while dry using a sterilized 18 mm diameter hollow steel punch. The coupons were placed in the previously prepared wells of the assay plate and hydrated for 2 minutes using 2 mL sterile saline (0.9% NaCl, Avantor), during which time they expanded (as expected) to cover the entire bottom of the well. The hydration solutions were then carefully aspirated, and the wells were immediately seeded with macrophages at a concentration of 3.55 × 10^5^ cells/well (2 mL of cell suspension per well). The positive control wells were seeded with the same number of cells and stimulated with 1 *μ*g/mL LPS for 24 h [36, 37]. The negative control (no treatment) was also seeded with the same number of cells, but only the RPMI culture medium was present in those wells. The plates were placed in a humidified incubator at 37°C, 5% CO_2_ for the desired time points. At the conclusion of the timepoints, the cell culture medium (supernatant) was recovered from each well, cellular debris wasremoved through centrifugation, and the supernatant was aliquoted and stored at −20°C.

### 2.2. Fibroblast Culturing and Seeding

Primary human dermal fibroblasts (adult (HDFa), ATCC PCS-201-012, Manassas, VA, USA) were cultured in a Fibroblast Basal Medium supplemented with a Serum-Free Fibroblast Growth Kit (ATCC). For the assay, fibroblasts were seeded for 24 hours in a 12-well tissue culture plate (assay plate) with the following treatments in triplicate (*n* = 3): incubation with the BWP, stimulation with LPS (positive control), and no treatment (negative control). An additional 72-hour time point for the BWP was assessed to determine if the BWP maintained its activity compared to itself at 24 hours. There were some indications in the literature of the effects the LPS stimulation could potentially have on the secretion of growth factors by fibroblasts, i.e., potential increase in tumor necrosis factor alpha levels, though the experimental set up was different than described here [38]. Nevertheless, this control treatment was used to maintain consistency with the positive control treatment of the macrophages.

The assay plate and the BWP were prepared as described in the previous section; however, 16 mm-diameter circular coupons of the BWP were used for this assay to allow for less material expansion within the wells. The seeding density for the fibroblasts was 2.5 × 10^5^ cells/well (2 mL of cell suspension per well). The plates were incubated at 37°C, 5% CO_2_ for the appropriate time points. At the designated time points, the cell culture medium (supernatant) was collected from each well, cellular debris was removed through centrifugation, and the supernatant was aliquoted and stored at −20°C.

### 2.3. Cytokine Array Screening Assay

Macrophage supernatants were analyzed via a cytokine array assay (Human XL Cytokine Array Kit ARY022B R&D Systems, Minneapolis, MN, USA) following the manufacturer's protocol. One membrane for each sample was blocked for 1 hour at room temperature on a rocking platform. The membranes were then incubated with the cell culture supernatants (0.5 mL) on the rocker at 4°C overnight. The membranes were washed three times with 1x wash buffer (provided by the manufacturer) and then incubated with detection antibody for 1 hour at room temperature on the rocking platform. Following three washes with the 1x wash buffer, streptavidin-HRP was added and the membranes were rocked at room temperature for 30 minutes. The membranes were washed again three times, followed by a one-minute incubation with the Chemi Reagent mixture. BioMax Light autoradiography films (Carestream, 1788207) were exposed to the membranes for the following timepoints: 1, 2, 4, 8, and 10 minutes. Multiple exposure times were used to obtain the optimal signal intensity. The films were then immediately developed using a GBX developer and fixer (Carestream, 5158613). Positive signals were identified using the transparency overlay provided in the kit, and analysis was performed by scanning the films with a transparency scanner and then determining the pixel intensity relative to the assay controls (reference spots) using ImageJ (National Institutes of Health, Bethesda, MD, USA). An initial run with 0.5 mL supernatant was performed to assess the level of biomarkers. If the signal intensities were low, a repeat run with increased supernatant was performed to obtain better resolution (increase the signal to background ratio) and determine if any additional biomarkers can be detected.

### 2.4. MMP and TIMP Assay

Macrophage supernatants were further analyzed via the Quantibody Human MMP Array 1 (RayBiotech QAH-MMP-1, Peachtree Corners, GA, USA). This assay allows the quantitative measurement of 10 human matrix metalloproteinase-related proteins MMP-1, MMP-2, MMP-3, MMP-8, MMP-9, MMP-10, MMP-13, TIMP-1, TIMP-2, and TIMP-4 with minimum levels of detection for the analytes between 0.02 and 0.1 ng/mL. The Quantibody array is a multiplexed sandwich enzyme-linked immunosorbent assay (ELISA) performed on a glass slide. For the assay, a sample from each well of the cell culture assay plate was evaluated in triplicate, thus utilizing nine individual arrays per treatment type. Because each protein is spotted in quadruplicate, there was an overall *n* = 36 for this assay.

The assay was performed according to the manufacturer's protocol. Briefly, the slides were removed from the packaging and allowed to dry for about 3 hours at room temperature prior to use. The wells were then blocked using 100 *μ*L of the sample diluent for 30 minutes at room temperature with gentle rocking. The assay protein standards were prepared by reconstituting the lyophilized protein mix and then performing a series of six 1 : 3 dilutions. Following the blocking period, the buffer was removed, and 100 *μ*L of either the samples or the protein standards was added to each well. To minimize background, the cell culture samples were diluted 1 : 2 with sample diluent prior to adding to the slides. The slides were incubated with the samples and standards overnight at 4°C on a rocking platform. The next morning, the slides were allowed to warm up to room temperature for 1 hour before proceeding to the next step. The samples were decanted, and the slides were washed five times with 150 *μ*L wash buffer I and two times with 150 *μ*L wash buffer II. Each wash was incubated for five minutes at room temperature on the rocker. After washing, the biotinylated detection antibody was added to each well at an amount of 80 *μ*L and the slides were rocked at room temperature for 2 hours. The slides were then washed as previously described. Next, 80 *μ*L of the Cy3 equivalent dye-conjugated streptavidin was added to each well, and the slides were incubated on the rocker at room temperature for 1 hour, protected from light. The slides were then washed five times with wash buffer I, as previously described. After washing, the gasket was removed from each slide and the slides were placed in a 4-slide holder/centrifuge tube. The tube was filled with 30 mL of wash buffer I and rocked at room temperature for 15 minutes. Wash buffer I was then decanted, 30 mL of wash buffer II was added, and the tube was rocked at room temperature for 5 minutes. Wash buffer II was decanted, and the slides were removed, rinsed gently with deionized water to remove any residue, and dried completely under a compressed nitrogen stream. The slides were then shipped to the manufacturer of the assay for scanning and data extraction.

The protein levels were determined by first normalizing the fluorescent signals to the internal controls on the arrays. Each signal was normalized using the following formula:

(1) $$\begin{array}{c} X\left(nY\right)=X\left(Y\right)\times \frac{P1}{P\left(Y\right)}, \end{array}$$

where *P*1 = the average signal density of the positive control spots on the reference array, *P(Y)* = the average signal density of the positive control spots on array *Y* (array being analyzed), *X(Y)* = the signal density for a particular spot on array for sample “*Y*”, and *X(nY)* = the normalized value for that particular spot “*X*” on array for sample “*Y*”.

As per the manufacturer's instructions, the reference array is to be defined by the researcher, and it was decided to use as the reference the array for each sample that had the highest average signal for the positive control spots. After normalization, the values from the blanks were subtracted, and the protein amounts were determined using the standard curves generated for each individual protein.

Statistical analysis was performed using JMP, Version 14 statistical software (SAS Institute Inc.) to determine significant differences at an apriori level of *P* < 0.05. Analysis of the data was based on a Kruskal–Wallis one-way analysis of variance on ranks and a Tukey–Kramer pairwise multiple comparison procedure. The results are presented in mean ± standard deviation (SD). Samples were run at least in triplicates (*n* = 3).

### 2.5. Growth Factor ELISA Assay

The Human Growth Factor ELISA Strip II kit with standards (Signosis, Santa Clara, CA, USA) was used to detect growth factors secreted by the fibroblasts. The plate consists of twelve strips containing 8 wells each, with each strip having a separate capture antibody per well. The assay allows for the detection of the following growth factors: basic fibroblast growth factor (FGFb), vascular endothelial growth factor (VEGF), epidermal growth factor (EGF), platelet-derived growth factor BB (PDGF-BB), beta-nerve growth factor (b-NGF), stem cell factor (SCF), tumor necrosis factor alpha (TNF*α*), and transforming growth factor beta (TGF*β*).

The assay was performed according to the manufacturer's instructions as follows. The standards were prepared in the assay plate by first diluting the indicated amount of the stock in diluent buffer in the first strip. A series of four two-fold serial dilutions was then performed. The resulting concentrations for the standards were: 4, 2, 1, and 0.5 ng/mL. The assay detection range was between 0.125 and 4 ng/mL. The samples were added in amounts of 100 *μ*L per well. One sample of cell culture supernatant was used per strip. Two control strips were also included, one strip was a blank, with 100 *μ*L diluent buffer per well, and the other was a control for the fibroblast basal medium, with 100 *μ*L per well of the medium that had not been used for cell culture. After adding the standards and samples, the plate was incubated at room temperature with gentle shaking (40 rpm) for 2 hours. The wells were then washed three times using 200 *μ*L of assay wash buffer per well. Following the washes, 100 *μ*L of the biotin-labeled antibody mixture was added to each well and the plate was incubated for 1 hour at room temperature with gentle shaking. The plate was washed three times as described previously, and then, 100 *μ*L streptavidin-HRP was added to each well. The plate was incubated with the streptavidin-HRP for 45 minutes at room temperature with gentle shaking. The plate was then washed three times. Next, 100 *μ*L of the substrate was added to each well. The substrate was incubated at room temperature for 20 minutes, and then, 50 *μ*L of the stop solution was added to each well. The stop solution was added row-wise, so that the reaction for each analyte was stopped at the same time. The absorbance was then read immediately at 450 nm. The data were blank corrected, and then, the total amount of each growth factor was determined using standard curves generated for each factor.

## 3. Results

### 3.1. Cytokine Array Screening Assay

**Figure 2:**
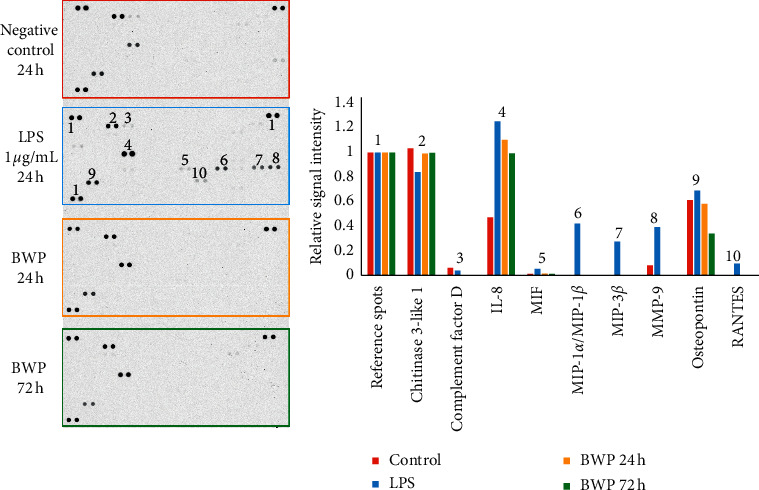
Cytokine array screening assay results. Left: scan of a 10-minute exposed film. Right: relative intensities for positive signals. Coordinates of positive signals are identified by the numbers on the LPS array.

**Figure 3:**
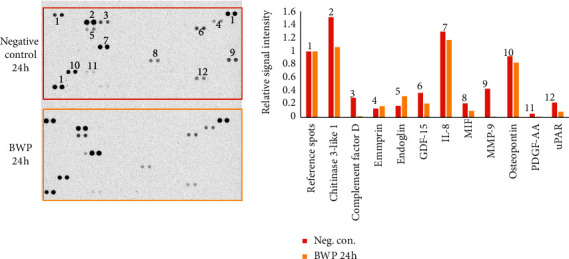
Cytokine array screening assay repeat results. Left: scan of a 4-minute exposed film. Right: relative intensities of positive signals. Coordinates of positive signals are identified by the numbers on the negative control array.

The results of the initial run of the cytokine array screening assay confirmed that the cells were responsive as biomarkers were detected for the LPS stimulated controls. Overall, the signal intensities were lower than desired. Therefore, the assay was repeated with a focus on the negative control and 24 h BWP exposure to obtain a better screening assessment of the initial cell response. Here, the amount of the cell culture supernatant was doubled (1 mL was used). The results of the initial and repeat runs are shown in Figures 2 and 3, respectively. A threshold of 5% of the reference signal was used for very faint signals.

### 3.2. MMP and TIMP Assay

**Figure 4:**
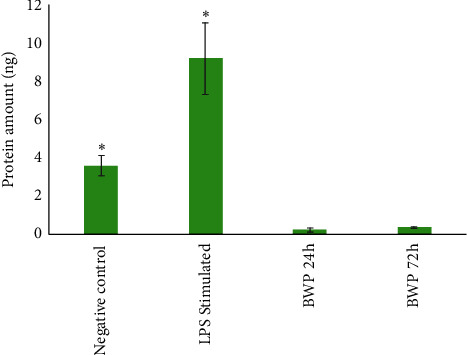
MMP-9 secreted by macrophages (3.55 × 10^5^ cells per sample) when cultured without treatment (negative control), 1 *µ*g/mL LPS (LPS Stimulated), or BWP for 24 and 72 h. ^*∗*^ denotes statistical significance (*P* < 0.01).

The highest amount of protein secretion for the controls was for TIMP-1 and MMP-9 (actual values and MMP-9/TIMP-1 ratio presented in Table 1). TIMP-1 was secreted in high amounts for the negative control and then in decreasing amounts for the LPS and BWP conditions. TIMP-2 also had similar results, but to a lesser extent. Of particular interest was MMP-9 as this protein was also detected in the cytokine array screening assay (Figure 4). MMP-9 was secreted in significantly higher (*P* < 0.01) amounts by the LPS-stimulated cells compared to the negative control and BWP samples. The negative control condition produced about a third less (still significantly lower, *P* < 0.01) MMP-9 than the LPS-stimulated cells. The BWP samples elicited significantly lower (negligible amounts, *P* < 0.01) secretion of MMP-9 compared to the LPS-stimulated and negative control samples. The BWP 24 h and 72 h were not statistically different (*P* < 0.05) from each other. All the levels detected were above the limit of detection for the assay.

### 3.3. Growth Factor ELISA Assay

**Figure 5:**
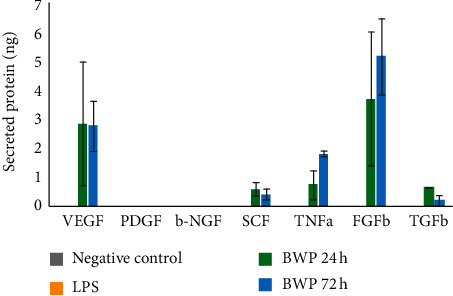
Levels of growth factors secreted by primary human dermal fibroblasts (2.5 × 10^5^ cells per sample). Assay detection range: 0.125–4 ng/mL.

The calculated growth factor levels are shown in Figure 5. All the levels detected were above the limit of detection for the assay. Both the negative control and LPS-stimulated samples did not produce a signal for any of the analytes, except for PDGF-BB, which had a very faint signal for the LPS samples. This, however, does not detract from the overall validity of the study as the samples were corrected for the blank, and cell culture medium and signals were observed for the BWP samples. The growth factor that was secreted in the greatest amounts was FGFb. The second most abundant factor was VEGF. PDGF-BB, despite showing a slight signal for the LPS samples, was not present at an amount quantifiable using the standard curve. EGF absorbance was detected; however, the standard curve for this growth factor was invalid and concentrations were not determined.

## 4. Discussion

### 4.1. Cytokine Array Screening Assay

Brief descriptions of the observed proteins are included in Table 2. The LPS-treated macrophages secreted several proteins that would be expected as part of a proinflammatory response. Elevated levels of interleukin-8 (IL-8), macrophage inflammatory proteins (MIP-1*α*/MIP-1*β* and MIP-3*β*), and MMP-9 were observed. The BWP also showed elevated IL-8, an important chemokine involved in the activation of neutrophils and other immune cells and associated with acute inflammation [54, 55]. However, there were no detectable macrophage inflammatory proteins or MMP-9 secreted from cells seeded on the BWP.

Comparing the signals seen in both Figures 2 and 3, it can be observed that, overall, the BWP profile is very similar to the negative control. The most notable results can be seen in Figure 3, in which the only major differences between the negative control and 24 h BWP samples were a lack of complement factor D, MMP-9, and platelet-derived growth factor-AA (PDGF-AA) in the BWP samples, as well as a decrease in the levels of growth/differentiation factor 15 (GDF-15), macrophage migration inhibitory factor (MIF), and most notably, urokinase-type plasminogen activator receptor (uPAR).

The data shown here indicate that the BWP has the potential to elicit desired biological effects on host inflammatory (macrophage) cells by not provoking a proinflammatory response. This study provided valuable *in vitro* insights but does not provide insight into temporal expression of the secretome, as a clinical study would provide. However, it does indicate what molecular targets should be further explored. As such, macrophage supernatants were further analyzed via a quantitative measurement of 10 human matrix metalloproteinase-related proteins.

### 4.2. MMP and TIMP Assay

In chronic wounds, highly elevated levels of proteases disrupt the balance between tissue breakdown and repair, which may lead to a stalled inflammatory phase and delayed healing. A relevant biomarker for wounds is MMP-9, which is one of the most abundant proteases in chronic wounds. Multiple clinical studies have analyzed fluid from chronic and acute wounds and concluded that increased MMP-9/TIMP-1 ratios are associated with poor wound healing [13, 16–19]. Therefore, diminishing degradation of growth factors and the extracellular matrix by reducing the MMP-9/TIMP-1 ratio is a viable wound management strategy to convert nonhealing wounds to a healing state.

The results from this preliminary study demonstrated that when macrophages interact with the BWP, secretion of MMP-9 significantly decreases (*P* < 0.01) and the MMP-9/TIMP-1 ratio is reduced by 5x and 16x compared to the negative and LPS stimulated controls, respectively (Table 1). The synthesis of multiple biomaterials to create the BWP may play a critical role in these results. The significant decrease of the MMP-9 activity could be attributed to the Manuka honey within the BWP since Manuka honey has been shown to suppress the expression of MMP-9 [28]. In addition to reducing the secretion of MMP-9 and the MMP-9/TIMP-1 ratio, the gelatin within the BWP can serve as a sacrificial substrate to buffer/reduce elevated levels of MMPs (including MMP-9) in the wound microenvironment [26]. Unlike collagen-based scaffolds that serve as a sacrificial substrate, this multimodal activity to control MMP-9 is a new approach in wound care that uniquely distinguishes the BWP.

A recent clinical study suggested that an MMP/TIMP ratio scoring system can serve as a potential predictive marker of diabetic foot ulcer healing, thus allowing an appropriate treatment plan to be developed [56]. The documented *in vitro* biological activity of the BWP reducing the MMP-9/TIMP-1 ratio suggests that it may be capable of instilling balance within the wound microenvironment, thereby impeding or disrupting chronic inflammation in wounds to advance the healing process.

### 4.3. Growth Factor ELISA Assay

Once balance to the wound microenvironment is restored, healing may progress out of the inflammatory phase and into the proliferative and remodeling phases, during which growth factors play key roles. Growth factors such as FGF, VEGF, EGF, TGF, PDGF, and others may accelerate wound healing through their physiological actions [57]. In chronic diabetic wounds, deficiencies in growth factors such as VEGF and TGF-*β* have been involved in the delayed healing rates [58]. Therefore, stimulating the body's innate response to secrete growth factors is a wound management strategy to progress wound healing. A brief description of the function of each growth factor detected in this in this study is included in Table 3.

The results from this preliminary study demonstrated that when fibroblasts interact with the BWP, secreted levels of FGFb, VEGF, TNF-*α*, TGF*β*, and SCF increased compared to the negative and LPS-stimulated controls. Per Table 3, these factors are known to promote cell differentiation, proliferation, migration, angiogenesis, and more. FGFb was observed at the highest amounts, which is of particular interest for wound healing considering FGFs have been shown to be more powerful angiogenic factors than VEGF and PDGF [67]. This stimulation of growth factor secretion could be attributed to each of the biomaterials synthesized to construct the BWP. Gelatin and Manuka honey are recognized for their biological activity with respect to wound healing. Specifically, Manuka honey has been shown to increase fibroblast activity and activate growth factors [27]. On the other hand, hydroxyapatite is a relatively unknown biomaterial in wound care. This inorganic mineral can bind a variety of proteins, ions, and other molecules from the surrounding environment which can trigger cell adhesion, proliferation, and differentiation [29, 30]. Based on the results of this study, HAp could play a role in the increased secretion of growth factors as it has been shown to promote wound healing via re-epithelialization, matrix formation, angiogenesis, and recruitment of fibroblasts [30–34].

The documented biological activity of the BWP in inducing secretion of key growth factors associated with wound healing by host cells *in vitro* suggests that the BWP may be capable of stimulating the body's innate response, including the release of essential growth factors, enabling the wound to advance toward the final stages of healing.

### 4.4. Future Studies

*In vitro* performance of the BWP may not be representative of clinical performance in human chronic wounds. Early clinical evaluations are underway, and further clinical trials are needed to support these findings. A follow-up study to this manuscript would be to compare the BWP to other wound products and to challenge the system with LPS primed or LPS + BWP conditions. Additional potential future *in vitro* studies include examining the role the BWP may play in the early and final stages of the wound healing cascade via hemostatic properties and collagen production, respectively. These future studies can provide insights into another fundamental strategy involving the BWP in wound management: its proactive use in acute wounds, such as diabetic and venous wounds early in the healing process, pressure ulcers, abrasions and surface wounds, traumatic injuries, donor site wounds, and surgical incision sites.

## Figures and Tables

**Table 1:** MMP-9 and TIMP-1 levels and corresponding ratios.

Treatment	MMP-9 (ng)	TIMP-1 (ng)	MMP-9/TIMP-1 ratio
Negative control	3.58	10.67	0.34
LPS stimulated	9.22	9.19	1.00
BWP 24 h	0.20	6.72	0.03
BWP 72 h	0.34	5.60	0.06

**Table 2:** Proteins detected using cytokine array assay.

Name	Description
CHI3L1	Chitinase 3-like 1; secreted by activated macrophages [39]
Complement factor D	Part of the alternative complement pathway [40]
Emmprin	Extracellular matrix metalloproteinase inducer; induces synthesis of several MMPs [41]
Endoglin	Transmembrane glycoprotein; expressed by monocytes transitioning to macrophages [42]
GDF-15	Growth/differentiation factor 15; stress responsive cytokine [43]
GRO*α*	Growth-regulated alpha protein; chemotactic for neutrophils [44]
IL-8	Interleukin-8; chemotactic for neutrophils [45]
MIF	Macrophage migration inhibitory factor; inflammatory cytokine [46]
MIP-1*α*/*β*	Macrophage inflammatory proteins; chemotactic and proinflammatory; produced by macrophages in response to bacterial endotoxins [47]
MIP-3*β*	Macrophage inflammatory protein 3 alpha; chemotactic [48]
MMP-9	Matrix metallopeptidase 9; involved in degradation of the extracellular matrix [49]
Osteopontin	Upregulated during macrophage differentiation; known to be induced in macrophages by inflammatory cytokines [50]
PDGF-AA	Platelet-derived growth factor; regulates cell growth and division; produced by macrophages in the wound healing stage [51]
RANTES	Regulated on activation, normal T cell expressed and secreted; chemotactic [52]
uPAR	Urokinase-type plasminogen activator receptor; role in plasminogen activation [53]

**Table 3:** Description of growth factors targeted in ELISA.

Name	Description
VEGF	Vascular endothelial growth factor; mediates vascular growth and angiogenesis [59]; encourages collagen deposition and epithelialization in wound healing [60]
EGF	Epidermal growth factor; mediates cellular proliferation, differentiation, and survival [61]
PDGF-BB	Platelet derived growth factor; mitogen for several cell types including fibroblasts and smooth muscle cells [62]
SCF	Stem cell factor; hematopoietic cytokine [63]
TNF*α*	Tumor necrosis factor alpha; acute inflammatory cytokine that plays a role in immunostimulation and resistance to infectious agents and tumors [57, 64]
FGFb	Basic fibroblast growth factor; important in the development and function of numerous organ systems. Some effects include stimulating smooth muscle cell growth, wound healing, and tissue repair [65]
TGF*β*	Transforming growth factor beta; multifunctional growth factor that plays important role in growth and development, inflammation and repair, and immunity [66]

## Data Availability

Data are available upon request at isaac@sweetbio.com.
